# Autophagy: mechanisms, roles in human diseases, and therapeutic perspectives

**DOI:** 10.3389/fcell.2026.1776289

**Published:** 2026-04-15

**Authors:** Jiazhen He, Teng Qi

**Affiliations:** 1 Shaanxi Eye Hospital, Xi’an People’s Hospital (Xi’an Fourth Hospital), Affiliated People’s Hospital of Northwest University, Xi’an, Shaanxi, China; 2 School of Medicine, The Chinese University of Hong Kong, Shenzhen, China

**Keywords:** autophagy, cancer, chaperone-mediated autophagy, human diseases, macroautophagy, mitophagy, neurodegenerative disorders, therapeutic targeting

## Abstract

Autophagy, a conserved intracellular degradation and recycling process, maintains cellular homeostasis by eliminating damaged organelles, misfolded proteins, and invading pathogens. Dysregulation of autophagy either excessive or insufficient contributes to the pathogenesis of numerous human diseases, spanning the respiratory, locomotor, circulatory, digestive, urinary, and nervous systems, as well as cancer. This Mini Review summarizes the core mechanisms and classification of autophagy, highlights its dual roles in various pathological conditions, discusses existing controversies and research gaps, and outlines potential future directions for therapeutic targeting. A concise overview of key findings provides readers with an updated understanding of autophagy’s multifaceted functions in disease development and treatment.

## 1 Introduction

Autophagy, derived from the Greek term meaning “self-eating,” was first morphologically described in 1966 and has since emerged as a critical physiological process in eukaryotic cells (De Duve and Wattiaux, 1966). It involves the sequestration of cellular cargo into membrane-bound structures (autophagosomes) that fuse with lysosomes for degradation and recycling of macromolecules, supporting cell survival under basal conditions and stress such as nutrient deprivation, hypoxia, oxidative stress (Osawa and Noda, 2019; Mizushima and Komatsu, 2011). Autophagy is orchestrated by autophagy-related genes (Atg) and regulated by key signaling pathways, including the mammalian target of rapamycin (mTOR) and adenosine monophosphate activated protein kinase (AMPK) (Hosokawa et al., 2009; Kim et al., 2011). While basal autophagy protects cells by maintaining homeostasis, its dysregulation disrupts cellular balance, leading to pathological states (Liu et al., 2023). Recent advances have revealed autophagy’s complex roles in human diseases, making it a promising target for therapeutic intervention (Malik et al., 2024). This review synthesizes current knowledge on autophagy’s mechanisms, disease associations, and future research directions.

## 2 Core mechanisms and classification of autophagy

### 2.1 Classification of autophagy

In mammals, autophagy is categorized into three primary types based on cargo delivery mechanisms: macroautophagy, microautophagy, and chaperone-mediated autophagy (CMA) (Crotzer and Blum, 2005; Parzych and Klionsky, 2014). Whether these three types coordinate or are interrelated remains unclear (Wang et al., 2023). This lack of clarity raises fundamental questions: For instance, when macroautophagy is pharmacologically inhibited, does the cell compensate by upregulating microautophagy or CMA to maintain proteostasis? Current literature lacks direct *in vivo* evidence mapping this compensatory crosstalk. One could hypothesize that the degree of compensation depends on stress intensity and cell type. In post-mitotic cells like neurons, where macroautophagy is constitutively active, a backup “parallel pathway” might be essential, whereas in dividing cells, dilution of damaged material via proliferation might reduce this dependency. Future studies utilizing triple-knockdown models for key genes in each pathway (e.g., ATG5 for macroautophagy, LAMP-2A for CMA) under specific stress conditions are needed to resolve this interrelationship. All three types of autophagy involve transporting cargo to lysosomes for degradation and recycling in mammals (Mizushima and Komatsu, 2011; Klionsky et al., 2008; Massey et al., 2006). However, the mechanisms of substrate delivery to the lysosome in these three types are different (Supplementary Table S1) (Yang and Klionsky, 2010).

Macroautophagy, the most extensively studied form, is often simply referred to as “autophagy” (Galluzzi and Green, 2019). It involves the formation of double-membraned autophagosomes that encapsulate cargo such as organelles, proteins and fuse with lysosomes. It is further divided into non-selective autophagy induced by nutrient deprivation, degrading bulk cytoplasm and selective autophagy targeting specific substrates like damaged mitochondria (mitophagy) or pathogens (xenophagy) (Yang and Klionsky, 2009; Orenstein et al., 2010). The core of both non-selective and selective autophagy is the formation of double-membrane autophagosomes (Orenstein et al., 2010; Shatz and Elazar, 2024).

Microautophagy occurs via direct invagination of lysosomal/vacuolar membranes to engulf cytoplasmic components, although it has received less attention (Kuchitsu and Taguchi, 2023). It does not involve the formation of double-membraned vesicles within the cytosol; instead, the lysosome itself directly engulfs intracellular material through inward invaginations of the lysosomal membrane or protrusions. Based on morphological changes in lysosomes in yeast and mammalian cells, three different types of microautophagy have been found: Type 1 (lysosome or vacuole protrusions), Type 2 (lysosome or vacuole invaginations), and Type 3 (with invaginations) (Oku and Sakai, 2018). It can also be classified as fission-type and fusion-type (Oku and Sakai, 2018; Li et al., 2012). The primary functions of microautophagy involve organelle remoulding, membrane homeostasis, metabolic adaptation and so on (Li et al., 2012; Schuck, 2020).

Chaperone-mediated autophagy (CMA) is currently reported only in mammalian cells and exhibits a high degree of specificity (Cuervo and Wong, 2014). It is an intracellular protein degeneration system within lysosomes, characterized by the involvement of heat shock protein family members that work together with lysosome-associated membrane protein 2A (LAMP-2A) located on the lysosomal membrane to transport cytosolic cargo to the lysosome for degradation. The distinguishing feature of CMA from macroautophagy and microautophagy is that it involves lysosome transport without the involvement of vesicles. Proteins from the cytoplasm can enter the lysosomal lumen by directly crossing the membrane (Kaushik and Cuervo, 2012).

Unlike microautophagy, CMA does not use membrane structures but relies on chaperone proteins to recognize cargo proteins that contain specific pentapeptide motifs (Ferreira et al., 2013). Then, these substrates unfold and are translocated individually directly across the lysosomal membrane. In comparison to CMA and microautophagy, macroautophagy forms double-membrane autophagosomes to sequester cargos and then carry them to the lysosome (Yang and Klionsky, 2009). Macroautophagy and microautophagy work well together in temporal patterns (Mejlvang et al., 2018; Mortimore et al., 1983),and naturally interconnect to regulate degradation processes in a compensatory manner (Caballero et al., 2021; Mesquita et al., 2021).

### 2.2 Key mechanisms of autophagy

**FIGURE 1:**
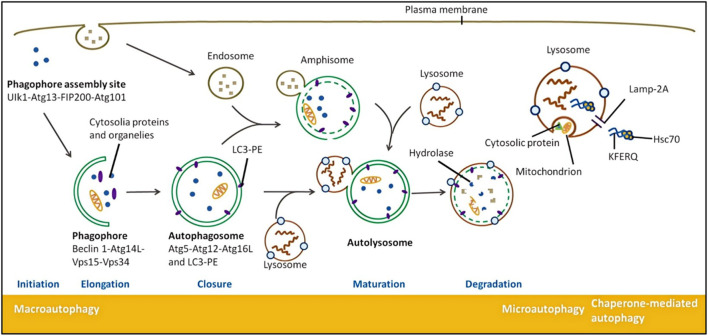
Schematic illustration of the complete molecular and cellular process of mammalian autophagy, including three major autophagic pathways.

Autophagy proceeds through four sequential steps: initiation, elongation, closure, and maturation/degradation (Figure 1). Initiation is triggered by stress signals that inhibit mTOR or activate AMPK, promoting the formation of the ULK1 complex (ULK1/2, Atg13, Atg101, FIP200) (Ganley et al., 2009; Itakura and Mizushima, 2010). AMPK inhibits mTORC1 complex formation, thereby derepressing the ULK1 complex and promoting autophagosome initiation (Meijer et al., 2015; Noda and Inagaki, 2015).

This diagram provides a detailed depiction of the complete autophagic process in cells, illustrating the sequential stages of autophagosome formation and maturation alongside the three major autophagic pathways. The process begins with autophagy induction at the phagophore assembly site near the plasma membrane, where the ULK1-Atg13-FIP200-Atg101 complex initiates signaling. During the autophagosome formation stage, the Beclin 1-Atg14L-Vps15-Vps34 complex regulates membrane nucleation on the phagophore (isolation membrane). Simultaneously, two ubiquitin-like conjugation systems drive membrane elongation: the Atg5-Atg12-Atg16L complex associates with the membrane, while LC3 is conjugated to phosphatidylethanolamine to form LC3-PE (LC3-II) and integrates into the expanding membrane. As the membrane elongates and bends, it ultimately closes to form a double-membraned autophagosome, encapsulating cytoplasmic contents such as proteins and organelles (e.g., mitochondria). During the maturation and fusion stage, the autophagosome fuses with endosomes to form an amphisome, which subsequently fuses with lysosomes—organelles containing diverse hydrolases—to create an autolysosome where encapsulated cargo is degraded and the breakdown products are recycled to the cytoplasm. In addition to this canonical macroautophagy pathway, the diagram depicts two other autophagy types: microautophagy, involving direct lysosomal membrane invagination to engulf small portions of cytoplasm; and chaperone-mediated autophagy, a highly selective process wherein cytosolic Hsc70 chaperone proteins recognize substrates containing KFERQ-like motifs and directly translocate them across the lysosomal membrane via specific receptors (labeled as lysosomal proteins in the diagram) for degradation.

Elongation of autophagic vesicles is driven by the Beclin-1/VPS34 complex, which generates phosphatidylinositol 3-phosphate (PI3P) to support membrane expansion (Itakura et al., 2008; Axe et al., 2008; Burman and Ktistakis, 2010; Proikas-Cezanne et al., 2015). Beclin1 is released by activated kinase JNK through phosphorylating BCL-2 and BIM, thereby disrupting the Beclin1/BCL-2 and Beclin1/BIM complexes. Free Beclin1 activates VPS34 to form a complex, and the resulting PI3P enhances the expansion of autophagic bodies.

Closure involves the ATG12-ATG5-ATG16L complex and lipidation of LC3 (LC3-I to LC3-II), which anchors to autophagosome membranes (Walczak and Martens, 2013; Tanida et al., 2004). ATG5-ATG12-ATG16L forms a polymeric complex through a series of interactions among ATG5, ATG12, and ATG16L, and then fuses with autophagic vesicles. After protein hydrolysis of the unbound and immature LC3 precursor protein (LC3-I) by ATG4 and binding to PE through ATG7 and ATG3, LC3-II is formed. Then, LC3 inserts into the phagophore membrane (Geng and Klionsky, 2008; Weidberg et al., 2011). The extending ends of phagophores fuse with each other to form a double-membrane-bound vesicle, known as an autophagosome, which encapsulates some cytoplasm along with proteins and organelle components (Yorimitsu and Klionsky, 2005). STX17 combines with SNAP29 and VAMP8 to form a SNARE complex, which transfers to the autophagosome membrane, enabling the fusion of autophagosomes with lysosomes to form autolysosomes (Yang and Klionsky, 2009). Within the autolysosomes, the hydrolytic proteases degrade cargo components (Lamb et al., 2013). Maturation occurs when autophagosomes fuse with lysosomes mediated by SNARE complexes like STX17-SNAP29-VAMP8 to form autolysosomes, where cargo is degraded by hydrolytic enzymes (Yang and Klionsky, 2009; Shen et al., 2021). STX17 undergoes deacetylation, with its C-terminal hairpin-like structure inserting into the complete autophagosome, interacting with SNAP29 and HOPS to promote the fusion of autophagosome with lysosome (Shen et al., 2021). Finally, the autophagosome membrane encapsulates and fuses with lysosomes containing hydrolytic enzymes and a low pH environment, allowing for the degradation of cargo (Lorincz and Juhasz, 2020). The resulting metabolites are recycled to sustain cellular metabolism (Yorimitsu and Klionsky, 2005).

Maturation occurs when autophagosomes fuse with lysosomes mediated by SNARE complexes like STX17-SNAP29-VAMP8 to form autolysosomes, where cargo is degraded by hydrolytic enzymes (Yang and Klionsky, 2009; Shen et al., 2021). STX17 undergoes deacetylation, with its C-terminal hairpin-like structure inserting into the complete autophagosome, interacting with SNAP29 and HOPS to promote the fusion of autophagosome with lysosome (Shen et al., 2021). Finally, the autophagosome membrane encapsulates and fuses with lysosomes containing hydrolytic enzymes and a low pH environment, allowing for the degradation of cargo (Lorincz and Juhasz, 2020). The resulting metabolites are recycled to sustain cellular metabolism (Yorimitsu and Klionsky, 2005).

### 2.3 Regulation of autophagy

**FIGURE 2:**
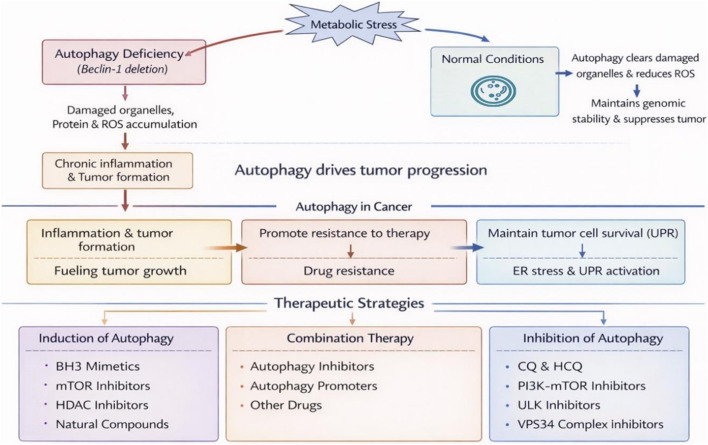
The dual context-dependent role of autophagy in cancer initiation and progression, and the corresponding targeted therapeutic strategies.

Autophagic flux is primarily regulated by mTOR and AMPK (Hosokawa et al., 2009; Kim et al., 2011). Under nutrient-rich conditions, mTOR inhibits the ULK1 complex, suppressing autophagy (Hosokawa et al., 2009). Conversely, stress such as starvation activates AMPK, which phosphorylates ULK1 and inhibits mTOR, triggering autophagy (Kim et al., 2011). AMPK is a sensor that measures cellular energy levels based on the AMP/ATP ratio, thus AMPK can be activated by an increase in intracellular AMP levels during starvation. Additional regulators include transcription factors such as TFEB, FOXO and signaling pathways such as PI3K-AKT, TGF-β, which fine-tune autophagy in response to cellular demands (Figure 2) (Ferreira et al., 2013; Fu et al., 2020).

This figure systematically elucidates the dual role of autophagy in cancer initiation and progression, along with corresponding therapeutic strategies. In normal cells or the early stages of tumor formation, autophagy suppresses tumorigenesis by clearing damaged organelles, protein aggregates, and reactive oxygen species (ROS), thereby maintaining genomic stability. Conversely, the absence of key autophagy genes (e.g., Beclin-1) leads to cumulative damage, chronic inflammation, and ultimately promotes tumorigenesis. However, in established tumors, autophagy undergoes a functional shift: endoplasmic reticulum stress (ER stress) and the unfolded protein response (UPR) in tumor cells activate autophagy, enabling it to provide nutrient recycling support for rapidly proliferating tumor cells, thereby sustaining their survival and driving tumor progression while inducing treatment resistance. Based on this dual role, therapeutic strategies are divided into two approaches: one involves inducing autophagy under specific conditions using BH3 mimetics, mTOR inhibitors, HDAC inhibitors, or natural compounds to suppress tumors; the other employs chloroquine (CQ), hydroxychloroquine (HCQ), or inhibitors targeting the PI3K-mTOR, ULK, or VPS34 complexes to block the autophagy flux when tumors rely on autophagy for survival. Ultimately, combining autophagy inducers or inhibitors with other therapies forms a synergistic treatment strategy aimed at more effectively intervening in cancer progression.

Under normal conditions, autophagy maintains genomic stability by eliminating damaged organelles and reducing reactive oxygen species, thereby exerting tumor-suppressive functions. Defects in autophagy (e.g., Beclin-1 deficiency) lead to damage accumulation, chronic inflammation, and tumorigenesis. However, after tumor formation, autophagy shifts to a pro-survival mechanism: it sustains tumor cell viability, promotes progression, and induces treatment resistance by responding to metabolic stress and endoplasmic reticulum stress. To address this paradox, therapeutic strategies are divided into two categories: one involves inducing autophagy (e.g., BH3 mimetics, mTOR inhibitors), while the other suppresses autophagy (e.g., chloroquine/hydroxychloroquine, ULK or VPS34 inhibitors), often in combination with other drugs to enhance efficacy.

## 3 Autophagy in human diseases

Supplementary Table S2 systematically summarizes the strategies and mechanisms by which related diseases exert therapeutic effects through regulating autophagy in different systems. In the digestive system, inhibiting autophagy (such as chloroquine combined with 5-FU) can enhance chemotherapy sensitivity in colon cancer (Selvakumaran et al., 2013; Sasaki et al., 2012); In the locomotor system, it exerts protective effects in osteoarthritis, osteoporosis, and sepsis muscle atrophy by enhancing selective autophagy (such as OPTN, PRKN), activating autophagy (such as TFEB, spermidine, trehalose), or inhibiting autophagy (such as Atg7 targeting, chloroquine), respectively (Slowicka et al., 2016; Ansari et al., 2018; Li et al., 2018; Sun et al., 2021; Lin et al., 2016). In the circulatory system, enhancing autophagy (such as overexpression of SIRT1 and Beclin 1) or regulating mitochondrial autophagy (such as BNIP3 and NIX) can improve heart failure and myocardial ischemia (Salminen and Kaarniranta, 2009; Hsu et al., 2008; Nishida et al., 2009; Hamacher-Brady et al., 2007; Gerald and Diwan, 2008); Inhibiting autophagy (such as AT1 receptor antagonists) can reduce myocardial cell death (Meyer et al., 2010). In the respiratory system, activating autophagy (such as mTORC1 inhibition, Beclin 1) helps to clear *Mycobacterium tuberculosis*, inhibit pulmonary arterial hypertension vascular remodeling, and lung cancer (Lam et al., 2012; Floto et al., 2007; Liu et al., 2011; Won et al., 2012); Restoring autophagy (such as IL-17A blockade) can alleviate pulmonary fibrosis (Mi et al., 2011). In the urinary system, activating autophagy (such as rapamycin) plays an anti-inflammatory, pathological improving, and autophagic regulatory role in interstitial cystitis, bladder outlet obstruction, and prostate cancer (Zhao et al., 2015; Schröder et al., 2013; Ding et al., 2020; Wadosky et al., 2019; Nguyen et al., 2014). In the nervous system, enhancing autophagy or lysosomal function (such as upregulation of Cathepsin, Rapamycin, Atg/Beclin 1) can clear A β and tau proteins in Alzheimer’s disease, alpha synuclein in Parkinson’s disease, and mutant Huntington’s protein (mHTT) in Huntington’s disease, and reduce neurotoxicity (Yang et al., 2011; Whyte et al., 2017; Liu and Li, 2019; Spencer et al., 2009; Yu et al., 2009; Ravikumar et al., 2004; Sarkar et al., 2007; Jeong et al., 2009; Qi et al., 2012). Autophagy plays a dual role in disease protective (e.g., clearing toxic aggregates in neurodegenerative disorders) or pathogenic (e.g., supporting tumor progression) (Mizushima and Komatsu, 2011; Burada et al., 2015).

### 3.1 Neurodegenerative diseases

In Alzheimer’s disease (AD), reduced Beclin-1 expression impairs autophagy, leading to amyloid-β (Aβ) and tau protein accumulation (Pickford et al., 2008; Keller et al., 2000). Enhancing autophagy via rapamycin or TFEB overexpression reduces protein aggregates and alleviates neurotoxicity (Liu and Li, 2019; Polito et al., 2014). In Parkinson’s disease (PD), mutations in PINK1 and PRKN (Parkin) disrupt mitophagy, promoting α-synuclein aggregation (Geisler et al., 2010; Michiorri et al., 2010). CMA dysfunction also contributes to α-synuclein accumulation in PD and amyotrophic lateral sclerosis (ALS) (Cuervo et al., 2004; Xilouri et al., 2009).

Notably, homozygous or compound heterozygous PINK1/PRKN mutations cause early-onset familial PD in some carriers, but heterozygous carriers or even some homozygotes may remain clinically unaffected, indicating incomplete penetrance and strong modifier effects (Krohn et al., 2020). Possible explanations include genetic background modifiers, compensatory mitochondrial quality control pathways, environmental exposures, and cell type–specific vulnerability; large population analyses show that heterozygous PINK1 variants are not uniformly high-penetrance risk factors, highlighting the need to study modifiers and cumulative stress exposures (Pickrell and Youle, 2015). This incomplete penetrance poses a critical question: What determines the “tipping point” from silent mutation to clinical disease? It is plausible that sub-threshold mitochondrial damage accumulates over decades. In asymptomatic carriers, residual PINK1/Parkin function or compensatory pathways (e.g., NIX/BNIP3-mediated mitophagy) may handle routine mitochondrial turnover. However, an “second hit”, such as a viral infection, environmental toxin, or aging-related decline in general proteostasis, could overwhelm this compensatory capacity, triggering a vicious cycle of mitophagy failure and α-synuclein aggregation. Experimentally validating this “multi-hit” hypothesis in induced pluripotent stem cell (iPSC)-derived neurons from asymptomatic versus symptomatic carriers under controlled stressors would be a logical next step. Beyond the “multi-hit” hypothesis, several fundamental questions remain: How do neurons integrate complex activation signals to fine-tune mitophagy? How do cells prioritize between different mitophagy initiation pathways (e.g., PINK1/Parkin-dependent vs. -independent)? Does a ‘parallel pathway’ exist that can be activated upon failure of the canonical PINK1/Parkin axis?

### 3.2 Cancer

Autophagy exhibits context-dependent roles in cancer. In early carcinogenesis, it suppresses tumor formation by clearing damaged DNA and ROS (Li et al., 2015). However, in advanced tumors, autophagy supports survival by providing nutrients and promoting drug resistance (Burada et al., 2015). For example, in colorectal cancer, autophagy inhibition with chloroquine enhances the efficacy of chemotherapy such as 5-fluorouracil (Selvakumaran et al., 2013; Sasaki et al., 2012). In lung cancer, Beclin-1 downregulation correlates with tumor progression, while ATG5 deficiency inhibits KRas(G12D)-driven tumorigenesis (Liu et al., 2011; Rao et al., 2014).

Mechanistic drivers of this context-dependent switch are multifactorial. Tumor-intrinsic metabolic stress (nutrient deprivation, hypoxia) and oncogene status (notably KRAS) increase autophagy dependency by creating demand for recycled metabolites (amino acids, lipids) that sustain mitochondrial metabolism and biosynthesis; hence RAS-driven tumours frequently become “autophagy-addicted” and use autophagy for survival under metabolic or therapeutic stress (Taucher et al., 2022). By contrast, intact tumor suppressors such as nuclear p53 can transcriptionally activate autophagy programs that remove damaged organelles and limit genomic instability, producing tumor-suppressive effects; loss, cytoplasmic mislocalization, or mutation of p53 can rewire this relationship so that autophagy instead supports survival of transformed cells (Shim et al., 2021). The mechanistic “switch” that converts autophagy from a tumor suppressor to a pro-survival mechanism remains a key gap. A critical question is whether this switch is binary or gradual. It is likely context-dependent: as the tumor microenvironment becomes more hypoxic and nutrient-depleted, the metabolic demand for autophagy-derived recycled substrates (amino acids, fatty acids) may outweigh the need for its quality-control function. This shift could be sensed by the AMPK/mTOR rheostat. One might hypothesize that a specific “metabolic threshold” (e.g., a defined ATP/ADP ratio or ROS level) dictates whether autophagy activation leads to cell survival or death. If this “survival threshold” exists, then a therapeutic corollary emerges: simply inhibiting autophagy in late-stage cancers might not be optimal; instead, we might need to “push” autophagy past a threshold that triggers autosis (autophagic cell death) rather than simply blocking it. Adding further complexity, microenvironmental hypoxia (HIF signaling) also promotes selective autophagy (e.g., BNIP3/NIX-mediated mitophagy) that reduces ROS and DNA damage, favouring survival under low-O_2_ niches and promoting therapy resistance. Thus, the “direction” of autophagy (protective vs. supportive) depends on oncogene/tumour suppressor genotype (e.g., KRAS/p53), metabolic and oxygenation state, and treatment-induced stress.

The breakthrough point in the future lies in analyzing the dynamic changes of autophagy in various stages of tumor occurrence, progression, metastasis, and drug resistance, as well as the “threshold” of autophagy in different tumor types, in order to achieve more precise drug delivery and precise regulation of tumor autophagy levels. Meanwhile, in the future, mechanism research should also be conducted to optimize the combination of autophagy regulators with chemotherapy, targeted therapy, and immunotherapy.

### 3.3 Cardiovascular diseases

In heart failure, excessive autophagy induced by Beclin-1 overexpression exacerbates cardiac hypertrophy (Zhu et al., 2007), while Sirt1-mediated autophagy protection mitigates oxidative stress (Salminen and Kaarniranta, 2009). Myocardial ischemia activates autophagy via AMPK, and targeting Bnip3/Nix improves ischemia-reperfusion injury (Hamacher-Brady et al., 2007; Matsui et al., 2007). This duality presents a therapeutic dilemma: How can we enhance the protective effects of mitophagy during ischemia without exacerbating the maladaptive, hypertrophic autophagy during reperfusion? The answer may lie in spatiotemporal regulation. It is conceivable that the molecular composition of autophagosomes differs between these states. For instance, ischemia-induced autophagy might selectively target damaged mitochondria (via BNIP3), while reperfusion-induced autophagy might non-selectively degrade essential cytoplasmic components. Therefore, rather than broadly inhibiting or activating autophagy, developing interventions that promote selective autophagy (e.g., enhancing the recognition of damaged mitochondria) while inhibiting bulk degradation could be a more precise strategy. Danon disease, caused by LAMP-2 mutations, is characterized by enhanced myocardial autophagy and cardiomyopathy (Tanaka et al., 2000).

### 3.4 Musculoskeletal diseases

Disrupted autophagy in bone cells (osteoblasts, osteoclasts) impairs bone remodeling, leading to osteoporosis and osteoarthritis (OA). In OA, PRKN-mediated mitophagy reduces chondrocyte ROS, while A2AR agonists prevent cartilage degradation (Ansari et al., 2018; Castro et al., 2020). Mutations in SQSTM1 (a selective autophagy receptor) cause Paget’s disease of bone, characterized by excessive autophagy (Laurin et al., 2002).

Future research can focus on the synergistic application of autophagy regulation with strategies such as anti-inflammatory, anti-apoptotic, and anti-ferroptosis, as well as the combined use of autophagy regulators with existing cardiovascular drugs such as statins and antiplatelet drugs.

### 3.5 Respiratory diseases

In pulmonary arterial hypertension (PAH), mTOR inhibition enhances autophagy, suppressing vascular proliferation (Krymskaya et al., 2011). Autophagy dysfunction contributes to idiopathic pulmonary fibrosis (IPF) via TGF-β1-mediated fibroblast activation (Patel et al., 2012), while in cystic fibrosis, Beclin-1 inactivation increases inflammation (Luciani et al., 2010).

Currently, there is a lack of validated autophagy related biomarkers to guide patient stratification. The detectable autophagy related molecules in the circulation, such as LAMP2A, require large-scale clinical validation. The discovery of LAMP2A as a common target for cross organ fibrosis suggests that other autophagy related factors may play common roles in various respiratory diseases (Jeong et al., 2009; Qi et al., 2012). Further research and systematic screening of these ‘cross disease targets’ are needed in the future.

### 3.6 Autophagy in aging, regeneration and wound healing

Autophagy has emerged as a central regulator of aging, tissue regeneration, and wound repair, processes that are closely interconnected with the onset and progression of human diseases. During aging, a progressive decline in autophagic activity contributes to the accumulation of damaged proteins and organelles, thereby impairing cellular homeostasis and promoting age-related pathologies. Restoration or enhancement of autophagy has been shown to improve cellular quality control, maintain stem cell function, and extend lifespan in multiple model systems. Notably, interventions such as caloric restriction and exercise have been demonstrated to activate autophagy through nutrient-sensing pathways, including inhibition of mTOR signaling, thereby contributing to healthy aging and longevity (Escobar et al., 2019; Chung and Chung, 2019).

In the context of tissue regeneration, autophagy plays a critical role in maintaining stem cell function and regenerative capacity. By removing damaged mitochondria and limiting oxidative stress, autophagy preserves stem cell self-renewal and differentiation potential (Bi et al., 2018). Impaired autophagic flux has been associated with stem cell exhaustion and reduced regenerative efficiency, whereas appropriate activation of autophagy can restore regenerative capacity in aging tissues (Bi et al., 2018).

Autophagy also exerts multifaceted and stage-dependent effects during wound healing. In the inflammatory phase, autophagy modulates immune responses and prevents excessive inflammation. During the proliferative phase, autophagy supports cell survival under hypoxic and oxidative stress conditions and promotes angiogenesis, keratinocyte migration, and re-epithelialization (Ren et al., 2022). In the remodeling phase, autophagy influences fibroblast activity and scar formation. However, dysregulated or excessive autophagy may impair healing, particularly in chronic wounds such as diabetic ulcers, highlighting the importance of precise regulation (Guo et al., 2016).

## 4 Discussion

### 4.1 Controversies and divergent perspectives

A key controversy in autophagy research is its dual role in cancer. Autophagy acts as a tumor suppressor in early carcinogenesis but supports cancer cell survival in advanced tumors (Burada et al., 2015; Ziparo et al., 2013). This duality complicates therapy, as inhibitors may help late-stage cancers but raise tumorigenic risks in normal cells. Another dispute is whether macroautophagy, microautophagy, and CMA function independently or compensate for one another (Wang et al., 2023; Caballero et al., 2021). In neurodegenerative diseases, enhanced macroautophagy may offset CMA dysfunction, yet supporting evidence is still insufficient.

Debate also surrounds mTOR inhibition in the nervous system. Rapamycin reduces protein aggregation and is neuroprotective in neurodegenerative models (Majumder et al., 2012; Singh et al., 2016), but mTORC1 supports synaptic plasticity and memory, and its inhibition can weaken long-term potentiation (Tang et al., 2002; Stoica et al., 2011). Effects depend on dosage, timing, and selectivity: moderate inhibition may improve proteostasis without blocking synaptic function, while sustained suppression disrupts metabolism and synaptic homeostasis (Majumder et al., 2012; Houde et al., 2010). Neuronal type, developmental stage, and disease state further shape outcomes (Tang et al., 2002; Stoica et al., 2011). Future therapies should separate autophagy activation from global translation repression via tailored dosing, selective mTORC1 targeting, or lysosomal biogenesis pathways. Comprehensive studies on flux, synaptic protein synthesis, and mTOR complex activity will help resolve these contradictions and support clinical translation.

### 4.2 Current research gaps

Despite significant progress, several gaps persist. First, the molecular mechanisms underlying selective autophagy such as mitophagy, ER-phagy in specific diseases are not fully elucidated. Second, biomarkers for monitoring autophagic flux in clinical settings are lacking, hindering the development of targeted therapies. Third, the role of autophagy in rare diseases such as Chediak-Higashi syndrome, Pompe disease is understudied (Zhang et al., 2010; Takikita et al., 2009a). Fourth, off-target effects of autophagy modulators such as rapamycin, chloroquine limit their clinical utility, and tissue-specific regulators remain to be identified.

### 4.3 Future directions

Future research should focus on four aspects. First, developing selective autophagy modulators that target disease-specific pathways such as mitophagy in PD, tumor-specific autophagy in cancer. Functional active peptides synthesized by computational and experimental methods are an important direction for the development of drugs related to autophagy pathway (Xie et al., 2025a; Xie et al., 2025b; Guan et al., 2025; Yao et al., 2025; Yao et al., 2024; Xie et al., 2010). Second, identifying robust biomarkers such as LC3-II/LC3-I ratio, SQSTM1/p62 levels to stratify patients for personalized therapy. Third, investigating the crosstalk between autophagy and other cellular pathways such as inflammation, apoptosis to design combinatorial treatments. Fourth, exploring autophagy’s role in emerging diseases such as COVID-19 where autophagy dysfunction exacerbates cytokine release (Zhao et al., 2016).

At the same time, emerging frontier technologies are rapidly transforming the landscape of autophagy research. Single cell multi omics and spatial transcriptomics now allow investigators to dissect autophagy regulation within heterogeneous tissues at unprecedented resolution, uncovering cell type specific metabolic and immune signatures that were previously masked in bulk analyses (Sundaram and Prabhu, 2026). Genome wide CRISPR Cas9 screening platforms further enable systematic identification of novel autophagy regulators and context dependent vulnerabilities, particularly in cancer and neurodegenerative disease models (DeJesus et al., 2016). At the same time, artificial intelligence driven protein structure prediction and drug discovery pipelines, exemplified by recent advances in deep learning based modeling, are accelerating the rational design of selective modulators targeting key nodes such as mTOR and ULK1 (Jumper et al., 2021). High resolution imaging approaches including cryo electron microscopy and live cell super resolution microscopy provide mechanistic insights into autophagosome initiation, membrane elongation, and cargo recognition at near atomic detail (Bhuin et al., 2025). Collectively, the integration of multi omics profiling, functional genomics, computational modeling, and advanced imaging is expected to refine mechanistic understanding and expedite the translation of autophagy targeted therapies into clinical practice. While these technologies are powerful, their limitations must be acknowledged. For example, single-cell sequencing provides a static snapshot, not a dynamic readout of autophagic flux. CRISPR screens often identify regulators under extreme conditions that may not reflect physiological reality. AI-driven drug design, while predictive, still struggles to accurately model the complex protein-lipid interactions crucial for autophagosome formation. Therefore, integrating these technologies is not just additive but necessary: computational predictions must be validated by high-resolution imaging, and multi-omics findings must be functionally tested via CRISPR in relevant disease models.

### 4.4 Therapeutic potential

Autophagy modulators are currently under clinical evaluation. Chloroquine and hydroxychloroquine, autophagy inhibitors, are combined with chemotherapy for cancer treatment (Selvakumaran et al., 2013), whereas the autophagy activator rapamycin demonstrates potential in neurodegenerative disorders (Liu and Li, 2019). Novel compounds including TFEB agonists and SQSTM1 modulators remain in preclinical stages, yet their clinical translation demands solutions for dose-related toxicity and tissue specificity.

Autophagy has advanced significantly in therapeutic development. Research has shifted from simple autophagy activation or inhibition to precise targeting of specific pathway nodes for functional reprogramming. For example, inhibiting PIKFYVE kinase redirects autophagic flux from degradation to secretion (Hung et al., 2023), while the Beclin1-SLC7A11 axis combines enhanced autophagy with ferroptosis (Yang et al., 2026), supporting functional modulation rather than simple flux control.

Rapamycin exerts neuroprotection via multiple targets, but its mTOR inhibition has dual effects. Although it facilitates harmful protein clearance, it may disrupt normal synaptic signaling. The conventional reliance on rapamycin and its analogs is evolving. KHS-101 activates TFEB through an mTOR- and calcineurin-independent mechanism, with blood-brain barrier permeability integrated into screening, directly resolving key barriers to central autophagy drug translation (Zhu et al., 2026).

Autophagy-targeted therapies have entered a new phase with improved precision. ULK1/2 inhibitor ULK-101 shows stronger autophagy inhibition than chloroquine in KRAS-driven lung cancer (Hinze et al., 2020); DCC-3116 is the first clinical-stage ULK1/2 inhibitor with favorable safety in RAS/RAF-mutated solid tumors (Khawaja et al., 2024). PIKFYVE inhibitor ipimod induces secretory autophagy to clear pathological proteins in ALS/FTD (Bhuin et al., 2025). KHS-101 activates TFEB via a novel pathway (Yang et al., 2026), and ZK824 induces autophagy through epigenetic regulation of TFE3/TFEB (Zhu et al., 2026).

Multimodal strategies are also emerging. The oral biomimetic probiotic peptide platform BTB Alg activates autophagy to exert anti-fibrotic effects in colitis (Yu et al., 2026). Radiation-modified naringin-functionalized gold nanoparticles regulate autophagy via the PI3K/Akt/mTOR pathway (ElBakary et al., 2026). The traditional Chinese medicine monomer indirubin modulates both autophagy and apoptosis (Xie et al., 2026).

In addition to the aforementioned strategies, an emerging therapeutic concept involves the sequential or combined use of autophagy inducers and inhibitors to achieve precise modulation of autophagic flux throughout different stages of tumor progression. This “smarter strategy” aims to harness the context-dependent nature of autophagy by first enhancing its tumor-suppressive functions or sensitizing cancer cells, followed by blockade of the pro-survival autophagic response during established disease or therapy resistance (Liu et al., 2020). Such a paradigm underscores the importance of dynamic monitoring of autophagic activity and personalized scheduling of autophagy-modulating agents in clinical settings.

### 4.5 Risks, off-target effects and clinical limitations

Clinically available autophagy modulators illustrate safety-efficacy tradeoffs. Lysosomotropic agents (chloroquine/hydroxychloroquine) inhibit autophagosome-lysosome fusion but carry dose-dependent ocular toxicity (retinopathy) and cardiac/neuromuscular risks with chronic use (Yusuf et al., 2023); systemic mTOR inhibitors (rapamycin/analogs) have known metabolic (hyperlipidaemia, insulin resistance), immunosuppressive and wound-healing adverse effects that limit chronic administration (Lin et al., 2022). These toxicities argue for strategies that (1) increase target specificity (e.g., selective ULK/VPS34/PIKFYVE inhibitors with tumour or tissue selectivity), (2) restrict exposure temporally (pulsatile dosing), or (3) combine lower-dose autophagy modulation with synergistic therapies (e.g., chemotherapy, targeted degraders) to reduce toxin burden. Clinical translation also requires robust pharmacodynamic biomarkers (noninvasive methods to measure autophagic flux) and tissue-penetrant agents for CNS diseases.

### 4.6 Non-pharmacological modulation of autophagy

In addition to pharmacological interventions, autophagy is profoundly influenced by non-pharmacological factors, which represent accessible and physiologically relevant strategies for disease prevention and health promotion. Among these, caloric restriction is one of the most extensively studied modulators of autophagy. It activates autophagic pathways primarily through inhibition of mTOR and activation of AMPK, thereby enhancing cellular quality control and promoting longevity. Importantly, the beneficial effects of caloric restriction on aging and metabolic health are at least partially dependent on autophagy activation (Ren et al., 2022).

Physical exercise is another potent inducer of autophagy across multiple tissues, including skeletal muscle, liver, and brain. Exercise-induced autophagy contributes to metabolic adaptation, mitochondrial quality control, and protection against age-related diseases. Notably, exercise and caloric restriction share overlapping molecular pathways, suggesting a convergent mechanism through which lifestyle interventions regulate autophagy and promote systemic health (Bi et al., 2018).

Dietary composition and metabolic status are key determinants of autophagic activity. Nutrient deprivation, intermittent fasting, and low-energy states activate autophagy primarily through AMPK activation and mTOR inhibition (Xinyan et al., 2025; Cozzo et al., 2020; Hofer et al., 2022). In contrast, nutrient abundance suppresses autophagy via activation of mTOR signaling (Cozzo et al., 2020; Hofer et al., 2022). Emerging evidence further indicates that specific dietary components and metabolic cues can fine-tune autophagic responses, thereby influencing disease susceptibility and progression (Cozzo et al., 2020).

In addition, various forms of cellular stress, including metabolic and oxidative stress, can modulate autophagy in a context-dependent manner (Hofer et al., 2022; Kroemer et al., 2010). Moderate stress often induces adaptive autophagy to maintain cellular homeostasis, whereas chronic or excessive stress may lead to dysregulated autophagic responses and contribute to pathological processes (Kroemer et al., 2010).

## 5 Conclusion

Autophagy is a multifaceted process with critical roles in health and disease. Its dysregulation contributes to a broad spectrum of pathological conditions, but targeted modulation offers therapeutic potential. On the base of some new technologies such as Endogenous fluorescence labeling technology, Live cell imaging, CRISPR gene editing technology and so on, resolving controversies, filling research gaps and developing selective modulators will advance our understanding and clinical application of autophagy-based therapies. Future studies should prioritize translational research to bridge preclinical findings with clinical practice, ultimately improving outcomes for patients with autophagy-related diseases.
