# Supplementary material for: Autophagy: mechanisms, roles in human diseases, and therapeutic perspectives
Source: Front Cell Dev Biol. 2026 Apr 15;14:1776289. doi: 10.3389/fcell.2026.1776289 (PMC13125100; doi:10.3389/fcell.2026.1776289)
Supplement: Supplementary file 1 [file Table1.docx]

**Supplementary materials**

**Supplementary Table 1.** Comparison of Three Types of Autophagy.

| **Feature** | **Macroautophagy** | **Microautophagy** | **Chaperone Mediated Autophagy**  **(CMA)** |
| --- | --- | --- | --- |
| Cargo selectivity | Bulk or selective | Mostly non selective | Highly selective |
| Cargo recognition | Receptors such as p62, NBR1 | Direct membrane invagination | KFERQ like motif recognized by HSC70 |
| Delivery mechanism | Double membrane autophagosome formation | Direct lysosomal membrane invagination | Direct translocation across lysosomal membrane |
| Vesicle formation | Yes | No | No |
| Key molecular components | ULK1 complex, Beclin 1, ATG proteins, LC3 | ESCRT related machinery | HSC70, LAMP 2A |
| Substrate type | Protein aggregates, organelles, pathogens | Cytosolic components | Soluble cytosolic proteins |
| Physiological role | Stress adaptation, organelle turnover | Membrane homeostasis, nutrient balance | Protein quality control |
| Representative diseases | Cancer, neurodegeneration, infection | Less well defined | Neurodegenerative diseases, aging |

**Supplementary Table 2.** The strategies and mechanisms of related diseases through regulating autophagy in different systems play therapeutic roles.

| **System** | **Disease** | **Target / Modulator** | **Autophagy Effect** | **Outcome** |
| --- | --- | --- | --- | --- |
| Digestive | Colon cancer | Chloroquine + 5-FU | Autophagy inhibition | Increased chemosensitivity |
| Locomotor | Osteoarthritis | OPTN | Enhanced selective autophagy | Reduced protein aggregation |
|  |  | PRKN overexpression | Mitophagy induction | Improved IL1B mediated OA |
|  |  | TFEB, spermidine, trehalose | Autophagy activation | Chondrocyte protection |
|  | Osteoporosis | Atg7 targeting, chloroquine | Autophagy suppression | Reduced bone loss |
|  |  | TRIM33 | PI3K AKT FOXO activation | Osteoblast protection |
|  | Sepsis induced muscle atrophy | TAK 242 | TLR4 NF κB inhibition | Reduced muscle loss |
| Circulatory | Heart failure | SIRT1 overexpression | Autophagy enhancement | Anti aging, oxidative resistance |
|  |  | Beclin 1 | Autophagy upregulation | Cardioprotection |
|  |  | AT1 receptor antagonists | Reduced autophagy mediated death | Cardiomyocyte protection |
|  | Myocardial ischemia | AMPK activation | Autophagy induction | Ischemic adaptation |
|  |  | BNIP3, NIX | Mitophagy activation | Therapeutic potential |
| Respiratory | Tuberculosis | mTORC1 inhibition | Autophagy activation | Reduced Mtb survival |
|  | PAH | mTORC1 blockade | Autophagy induction | Reduced vascular proliferation |
|  | IPF | IL 17A blockade | Restored autophagy | Reduced fibrosis |
|  | Lung cancer | Beclin 1 | Autophagy activation | Tumor suppression correlation |
| Urinary | IC BPS | Rapamycin | Autophagy activation | Reduced inflammation |
|  | BOO | Rapamycin | Autophagy induction | Improved bladder pathology |
|  | Prostate cancer | AR inhibition | AMPK activation mTOR inhibition | Regulated autophagy response |
| Nervous | Alzheimer’s disease | Cathepsin activation | Lysosomal enhancement | Reduced Aβ and tau toxicity |
|  |  | Rapamycin | Autophagy activation | Tau clearance |
|  | Parkinson’s disease | Atg, Beclin 1 upregulation | Autophagy activation | α synuclein clearance |
|  | Huntington’s disease | Rapamycin, CCI 779 | mTOR inhibition | mHTT clearance |
|  |  | HSC70, LAMP2A | CMA enhancement | Reduced neurotoxicity |
